# Acute hypoxia increases the cerebral metabolic rate – a magnetic resonance imaging study

**DOI:** 10.1177/0271678X15606460

**Published:** 2015-10-02

**Authors:** Mark B Vestergaard, Ulrich Lindberg, Niels Jacob Aachmann-Andersen, Kristian Lisbjerg, Søren Just Christensen, Ian Law, Peter Rasmussen, Niels V Olsen, Henrik BW Larsson

**Affiliations:** 1 Functional Imaging Unit, Department of Clinical Physiology, Nuclear Medicine and PET, Rigshospitalet, Glostrup, Denmark; 2 Department of Neuroscience and Pharmacology, The Faculty of Health Sciences, University of Copenhagen, Copenhagen, Denmark; 3 Institute of Clinical Medicine, The Faculty of Health Sciences, University of Copenhagen, Copenhagen, Denmark; 4 Department of Clinical Physiology, Nuclear Medicine and PET, Rigshospitalet, Copenhagen, Denmark; 5 Department of Neuroanaesthesia, The Neuroscience Centre, Rigshospitalet, Copenhagen, Denmark

**Keywords:** Cerebral blood flow, cerebral hemodynamics, energy metabolism, high altitude, MR spectroscopy, magnetic resonance imaging

## Abstract

The aim of the present study was to examine changes in cerebral metabolism by magnetic resonance imaging of healthy subjects during inhalation of 10% O_2_ hypoxic air. Hypoxic exposure elevates cerebral perfusion, but its effect on energy metabolism has been less investigated. Magnetic resonance imaging techniques were used to measure global cerebral blood flow and the venous oxygen saturation in the sagittal sinus. Global cerebral metabolic rate of oxygen was quantified from cerebral blood flow and arteriovenous oxygen saturation difference. Concentrations of lactate, glutamate, N-acetylaspartate, creatine and phosphocreatine were measured in the visual cortex by magnetic resonance spectroscopy. Twenty-three young healthy males were scanned for 60 min during normoxia, followed by 40 min of breathing hypoxic air. Inhalation of hypoxic air resulted in an increase in cerebral blood flow of 15.5% (*p* = 0.058), and an increase in cerebral metabolic rate of oxygen of 8.5% (*p* = 0.035). Cerebral lactate concentration increased by 180.3% (p<10-6), glutamate increased by 4.7% (p<10-4) and creatine and phosphocreatine decreased by 15.2% (*p*<10-3). The N-acetylaspartate concentration was unchanged (*p* = 0.36). In conclusion, acute hypoxia in healthy subjects increased perfusion and metabolic rate, which could represent an increase in neuronal activity. We conclude that marked changes in brain homeostasis occur in the healthy human brain during exposure to acute hypoxia.

## Introduction

Normal brain function requires a stable microenvironment with regard to pH, oxygen and glucose delivery. The brain has a number of adaptive mechanisms to keep this environment relatively stable even with major changes in external factors.

In this study, we investigated the effect of a poikilocapnic hypoxic challenge (10% fraction of inspired O_2_ (FiO_2_)) on the cerebral energy metabolism in 23 young healthy males using non-invasive magnetic resonance imaging (MRI). In poikilocapnic hypoxia the CO_2_ blood content is not controlled but fluctuate freely and will decrease during hypoxia due to increased ventilation, contrary to isocapnic hypoxia where CO_2_ is added to the hypoxic air to account for wash out of CO_2_ from increased ventilation.

Regulation of brain perfusion is one of the most important adaptive mechanisms employed to secure the brain during various circumstances. In acute hypoxia, increased brain perfusion compensates for reduction of the delivery of oxygen from the blood. Kety and Schmidt^1^ found a 35% increase in global cerebral blood flow (CBF) during inhalation of air with a 10% fraction of O_2_ (FiO_2_) but found no significant change in the global cerebral metabolic rate of oxygen (CMRO_2_), despite definite mental changes.

Whereas the CBF increase during hypoxic exposure is well known,^2^ uncertainty exists regarding the concomitant change in CMRO_2_. New MRI methods allow non-invasive measurement of mean CMRO_2_, i.e. the brain’s total oxygen consumption divided by the total brain volume, based on measurement of the concentration of deoxyhemoglobin in the sagittal sinus combined with measurement of total blood flow to the brain and Fick’s principle. Using such a technique, two studies have reported a slight but significant increase in CMRO_2_ during a hypoxic challenge.^3,4^ On the contrary, studies using Kety-Schmidt techniques and blood samples found no change in CMRO_2_.^1,5,6^

Cerebral lactate release increases during hypoxia, and lactate contributes up to 9% of total cerebral energy turnover during hypoxia.^7^ Through the use of two-photon laser-scanning microscopy in rat brain slices, it has been shown that hypoxic O_2_ levels enhance the glycolytic rate of the brain.^8^ Concomitantly, astrocyte-mediated vasodilation is mediated by extracellular lactate and prostaglandin E2 (PGE2) accumulation.^9^ In addition, lactate, via its action on a specific lactate receptor (GPR81), may inhibit cAMP generation, causing a slowing of glycolytic rate when the lactate concentration rises.^10^ Furthermore, lactate may act as a volume transmitter, regulating CBF and the brain energy turnover of large ensembles of neurons.^11^ It is also known that severe ischemia, as observed in stroke, results in local release of the excitatory neurotransmitter glutamate. This glutamate release, which constitutes an excitotoxic mechanism, enhances neural activity and is detrimental for tissue survival.

During hypoxia, ventilation rate will increase and cause hypocapnia, which also has an effect on the cerebral metabolism. However, the reported effect of hypocapnia is also diverse. A study on patients with cerebral vascular disease showed no conclusive result,^12^ and a study of healthy humans reported a small but non-significant increase in CMRO_2_ during hypocapnia.^13^

In this study, we used magnetic resonance proton spectroscopy (MRS) to investigate whether hypoxia-induced changes in CBF and CMRO_2_ are related to concomitant changes in cerebral lactate, creatine (Cr) and glutamate levels. By MRS it is possible to concurrently measure lactate, Cr, N-acetylaspartate (NAA) and glutamate concentration non-invasively. We expect increased cerebral lactate production if the oxygen delivery to the brain is sufficiently reduced during hypoxia. Phosphocreatine (PCr) and Cr work as energy reserve as ATP can rapidly be produced from creatine kinase. Glutamate is an excitatory neurotransmitter and is associated with neuronal activity. Measurements of lactate, Cr and glutamate along with CBF and CMRO_2_ make it possible to get a comprehensive picture of the cerebral metabolism non-invasively.

Based on published results, we expected to find no change or a slight increase in CMRO_2_ and an increase in lactate concentration. The effect on glutamate and Cr is not known, although we suspect a possible interaction between energy metabolism and these metabolites.

## Methods

We included 23 young, healthy males (mean age: 27.4 years, range: 18–40 years). The study was conducted as part of a larger project investigating the effect of erythropoietin on the cerebral metabolism. The study was approved by the Danish National Committee on Health Research Ethics (h-4-2012-167) and was conducted according to the Declaration of Helsinki. All subjects gave written informed consent prior to participation.

All scans were performed on a Philips 3T Achieva MRI scanner (Philips Medical Systems, Best, The Netherlands) using a 32-channel phase array head coil.

The subjects were scanned during normoxia for approximately 60 min, followed by a 40 min hypoxia period. During hypoxia, the subjects were fitted with a mouthpiece connected by a reservoir balloon to a gas cylinder delivering 10% O_2_ in nitrogen. No rebreathing was possible. The MR-compatible system did not allow us to control the level of CO_2_.

Arterial blood gas was sampled through an arterial catheter inserted in the radial artery to monitor arterial oxygen saturation (SaO_2_). If the arterial saturation fell under 60%, the subjects were asked to increase their breathing rate for safety reasons. Samples were drawn immediately before switching to hypoxia; seven times during hypoxia at 1, 2, 3, 5, 10, 20 and 32 min after the start of hypoxia; and once 3 min after hypoxia, during recovery. Immediately after they were taken, the samples were analyzed for SaO_2_, O_2_ pressure (PaO_2_), CO_2_ pressure (PaCO_2_), hematocrit, glucose, hemoglobin and lactate concentrations by blood gas analysis (Radiometer ABL800 Flex, Radiometer, Copenhagen, Denmark). Arterial oxygen concentration (CaO_2_) was calculated from SaO_2_, PaO_2_ and hemoglobin levels and includes both the freely diluted oxygen and the oxygen bound to hemoglobin. Measurements at 20 and 32 min were carefully temporally matched with MRI measurements of MRS and CMRO_2_. The effect of hypoxia on the blood content presented in the result section and Table 1 is from blood samples taken after approximately 32 min of hypoxia.

**Table 1.** Summary of the results and statistics.

	Normoxia	Hypoxia	Absolute change	Percent change	T-test *p* value	Regression *p* value
Blood content						
CaO_2_ (mmol/L)	8.5±0.6	6.8±1.3	–1.7	–20.5	<10-6 ^a^	
PaO_2_ (kPa)	14.1± 1.4	5.3± 1.3	–8.8	–62.2	<10-6 ^a^	<10-6 ^a^
SaO_2_ (%)	98.0± 0.5	78.4± 11.2	–19.6	–20.0	<10-6 ^a^	<10-6 ^a^
SvO_2_ (%)	68.5±7.6	48.7±11.0	–19.9	–29.0	<10-6 ^a^	<10-6 ^a^
PaCO_2_ (kPa)	5.5±0.5	4.1±0.8	–1.3	–24.3	0.002^a^	0.18^a^
Hemoglobin (mmol/L)	8.7±0.6	8.8±0.6	0.1	1.2	0.035^a^	0.053
Glucose (mmol/L)	5.9±0.6	5.4±0.4	–0.5	–8.2	<10-4 ^a^	0.014^a^
Blood lactate (mmol/L)	1.0±0.2	1.0±0.4	0.05	4.9	0.38	0.089
Hemodynamics						
Heart Rate (bpm)	57.0±9.0	69.0±10.2	11.9	20.9	<10-6 ^a^	<10-6 ^a^
MAP BP (mmHg)	76.6 ±8.4	75.1±8.2	–1.5	–0.02	0.17	0.024
Cerebral metabolism						
CDO_2_ (µmol/100 g/min)	494.3±54.7	433.2±99.8	–61.1	–12.4	0.01^a^	0.73
CBF (mL/100 g/min)	58.2±6.5	67.2±22.8	9.0	15.5	0.058	<10-6 ^a^
A-V O_2_ (%)	29.5±7.7	29.8 ±10.3	0.0	0.9	0.89	0.095
CMRO_2_ (µmol/100 g/min)	148.9± 40.3	161.6± 53.7	12.7	8.5	0.035^a^	0.004^a^
NAA (mmol/L)	7.9±0.4	7.8±0.7	–0.1	–1.4	0.36	0.18
Glutamate (mmol/L)	6.4±0.4	6.7±0.5	0.3	4.7	<10-4 ^a^	0.004^a^
Cerebral lactate (mmol/L)	0.4±0.2	1.2±0.3	0.8	180.3	<10-6 ^a^	<10-6 ^a^
tCr (mmol/L)	3.1 ±0.3	2.6± 0.6	–0.5	–15.2	<10-3 ^a^	0.002^a^

a A statistically significant change (*p* < 0.05).

CaO_2_: arterial O_2_ concentration; PaO_2_: arterial O_2_ pressure; SaO_2_: arterial saturation; SvO_2_: venous saturation; PaCO_2_: arterial CO_2_ pressure; MAP BP: mean arterial blood pressure; CDO_2_: cerebral O_2_ delivery; CBF: cerebral blood flow; A-V O_2_: arteriovenous O_2_ saturation difference; CMRO_2_: cerebral metabolic rate of O_2_; NAA: N-acetylaspartate; tCr: creatine and phosphocreatine.

The arterial saturation was also measured continuously by pulse oximetry. Electrocardiography was recorded during the whole scan with the MRI scanner’s accessory hardware.

### MRI protocol

MRI methods were used to estimate global mean CBF, CMRO_2_ and regional metabolite concentrations in the visual cortex.

CMRO_2_ can be computed by Fick’s Principle:

$${\mathrm{CMRO}}_{2}=C_{a}\cdot \mathrm{CBF}\cdot ({\mathrm{SaO}}_{2}-{\mathrm{SvO}}_{2})(1)$$

where SaO_2_ and SvO_2_ are the arterial and venous oxygen saturation, respectively, and *C_a_* is the oxygen concentration (mmol/L) and was calculated for each subject from the hemoglobin concentration. The arterial saturation was measured by blood sampling and the venous saturation was measured in the sagittal sinus by susceptometry-based MRI imaging (SBO).

CBF was measured by phase-mapping techniques in the internal carotid arteries and in the vertebral arteries. CBF and SvO_2_, from which CMRO_2_ is quantified, were measured during normoxia and after approximately 32 min of hypoxia. SvO_2_ was also measured during the transition from normoxia to hypoxia over a period of 3 min.

Metabolite concentrations were measured in the visual cortex by proton MRS. MRS was acquired over 12 min during normoxia and again after approximately 20 min hypoxia.

Anatomical scans were recorded to estimate brain size for normalizing functional data. Brain mass was estimated from grey and white volume segmented by FSL (FMRIB Software Library, Oxford University, Oxford, UK) functions (FAST) and assuming brain density to 1.05 g/mL.

**Figure 1.**
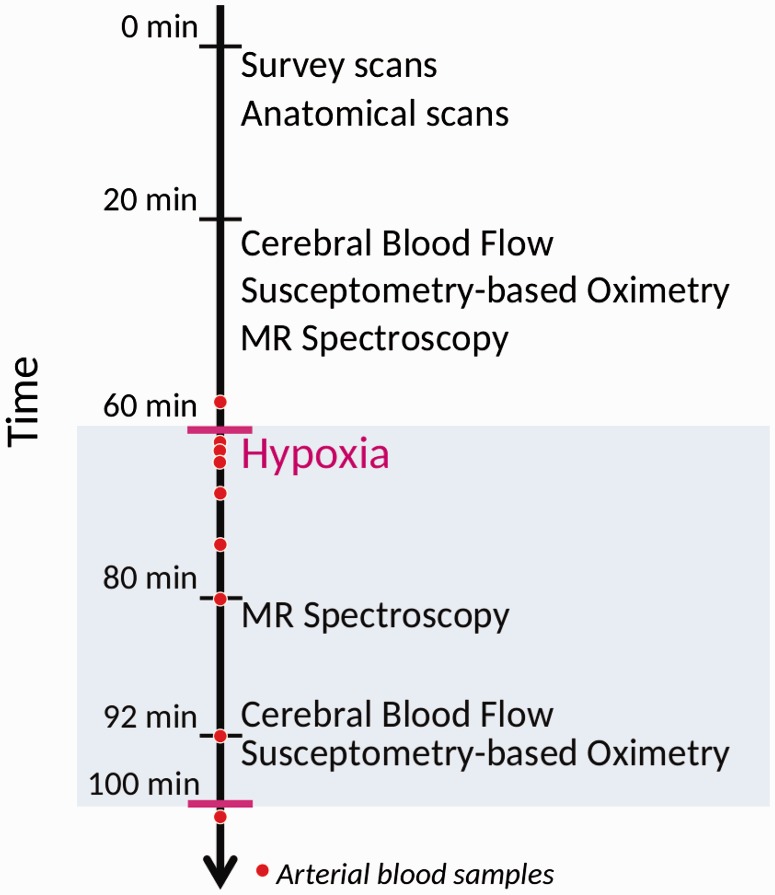
Timing of the measurement of parameters during MRI. Survey scans, anatomical measurements, cerebral blood flow, susceptometry-based oximetry and MR spectroscopy were recorded during the initial 60 min of normoxia. After 60 min, the subjects started to inhale 10% FiO_2_ hypoxic air. After 20 min of hypoxic exposure, the MR spectroscopy measurements were repeated. After 32 min, the recording of CBF and SBO was repeated under hypoxic exposure.

The timing of the MRI measurements can be seen in Figure 1.

### CMRO_2_

Recently, new MRI techniques have been developed to measure the oxygen saturation of the blood leaving the brain in the sagittal sinus. This, in combination with measurements of CBF, makes it possible to calculate CMRO_2_ using Fick’s Principle (equation 1).

**Figure 2.**
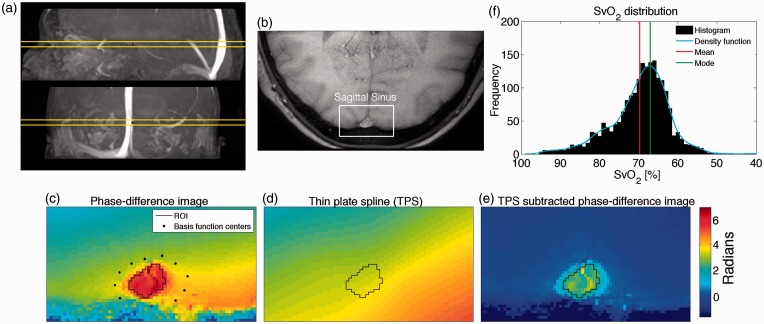
(a) Sagittal and coronal view of angiography highlighting the sagittal sinus with the imaging plane visualized. (b) Part of brain covering the sagittal sinus used for calculating SvO_2_. (c) Phase-difference map with region of interest (ROI) in the sagittal sinus and basis function centers located in surrounding tissue used for calculating thin plate spline model (TPS). (d) Calculated TPS model. (e) Phase-difference map corrected with TPS. The field inhomogeneity is rectified and the tissue surrounding the sagittal sinus is now close to zero. (f) Example of distribution of SvO_2_–values from each voxel from the 10 repeated measurements in the ROI for one subject. The mode of this distribution is slightly higher than the mean.

In this study, we used susceptometry-based oximetry.^14^ Susceptibility maps (ϕ) were created with a dual-echo gradient-echo sequence (1 slice, FOV=224×176mm2, voxelsize=0.5×0.5×5mm3, TE1=7.00ms, TE2=20.29ms, flipangle=30∘, 10 repeated measures) and modulus, real and imaginary complex values from both echoes were saved. The imaging slice was placed orthogonal to part of the sagittal sinus parallel to the B0-magnetic field (Figure 2(a)).

Susceptibility maps were computed by subtracting phase-value maps corresponding to the two images generated with the short- and long-echo times. The susceptibility maps were automatically unwrapped as described by Goldstein et al.^15^ Further unwrapping was performed manually if necessary.

SBO utilizes the difference in magnetic susceptibility between intravascular blood and the surrounding tissue to estimate saturation. The surrounding tissue is assumed to have the same susceptibility as fully oxygenated arterial blood. The difference in susceptibility can be related to saturation by an exact theoretical equation, where blood vessels are modeled as long paramagnetic cylinders located parallel with the static magnetic field, as described in equation (2)^16^

(2) $${\mathrm{SvO}}_{2}=(1-\frac{2|\phi_{\mathrm{ss}}(\bar{x})-\phi_{\mathrm{tissue}}(\bar{y})|}{\Delta \mathrm{TE}\gamma \Delta \chi_{\mathrm{do}}B_{0}(\cos2\theta -\frac{1}{3})\mathrm{Hct}}+\frac{\Delta \chi_{\mathrm{oxy}}}{\Delta \chi_{\mathrm{do}}})\times 100$$

where $\phi_{\mathrm{ss}}(\bar{x})$ represents the phase values from the sagittal sinus, $\phi_{\mathrm{tissue}}(\bar{y})$ represents the phase values from the surrounding tissue, θ is the angle between the sagittal sinus and the B0 field, Hct is the hematocrit level, $\Delta \chi_{\mathrm{do}}$ is the susceptibility difference between fully deoxygenated blood and fully oxygenated blood and $\Delta \chi_{\mathrm{oxy}}$ is the susceptibility difference between fully oxygenated erythrocytes and water. Different values for $\Delta \chi_{\mathrm{do}}$ have been suggested, ranging from 4π·0.18ppm^17^ to 4π·0.27ppm.^18^ This study used $\Delta \chi_{\mathrm{do}}=4\pi \cdot 0.27\text{ppm}$ and$\Delta \chi_{\mathrm{oxy}}=4\pi \cdot (-0.008)\text{ppm}$ as suggested by investigations of freshly drawn human blood.^19^

The difference in susceptibility between venous blood and tissue has previously been calculated by drawing a region of interest (ROI) in the sagittal sinus and in the surrounding tissue. The surrounding tissue has uniform susceptibility and therefore should ideally have the same phase values. However, because of magnetic field inhomogeneities, this is not the case. The phase values in the tissue ROI therefore depend on its location. Previously, this has been addressed by fitting a second-order polynomial plane model to the tissue and subtracting the plane to correct for inhomogeneities or by high-pass filtering to remove the low-frequency variation in inhomogeneity.^20^ This study applied a thin-plate spline model (TPS)^21^ to fit the tissue phase values. TPS is more flexible than a second-order polynomial and can correct for larger inhomogeneities. Control points from the tissue surrounding the sagittal sinus were used to compute the TPS. By interpolating the TPS values to cover the sagittal sinus, the susceptibility difference between venous blood and tissue, $|\phi_{\mathrm{ss}}(\bar{x})-\phi_{\mathrm{tissue}}(\bar{y})|$ can be found by subtracting the interpolated values for the same ROI from the thin-plate spline model from the phase values in the ROI covering the sagittal sinus, $|\phi_{\mathrm{ss}}(\bar{x})-\phi_{\mathrm{tps}}(\bar{x})|$. This process eliminates the need to draw a tissue ROI and concurrently corrects for field inhomogeneities. Example of correction by TPS is illustrated in Figure 2(c–e).

The value that appeared most often, the mode, in the inhomogeneity-corrected phase values of all the voxels in the ROI covering the sagittal sinus was found and used to calculate the oxygen saturation. The mode is used rather than the mean because the values might not be normally distributed as a result of partial-volume contamination. Voxels at the edge of the sagittal sinus will contain tissue from outside the sagittal sinus, which will reduce the susceptibility of the voxel compared to voxels containing only blood. This will skew the distribution and affect the mean of the values. However, the mode will be unaffected by this skewing of the distribution. An example of the distribution of SvO_2_–values calculated from voxel in the ROI with the mean and the mode is illustrated on Figure 2(f). If there is no partial-volume contamination, the values will be normally distributed and the mean and mode will be equal. The mode was calculated by fitting a kernel density function to the distribution of the phase values of all the voxels in the ROI from the 10 repeated measures.

All processing was performed using Matlab (Mathworks, Natick, MA, USA) scripts developed in house.

### Total cerebral blood flow

The global mean CBF was calculated by measuring the velocity in the carotid and vertebral arteries using a phase-encoding technique (1 slice, FOV=240×240mm2, voxelsize=0.75×0.75×8mm3, TE=7.33ms, TR=27.63ms, flipangle=10∘, 10 repeated measures, non-gated, velocityencoding=100cm/s).

The imaging slice was placed orthogonal to the arteries. CBF was calculated by measuring the mean velocity and cross-sectional area of each of the four feeding arteries. Processing was performed using in-house Matlab scripts.

The cerebral delivery of O_2_ (CDO_2_) was calculated by multiplying CBF by the arterial oxygen concentration.

### Magnetic resonance spectroscopy

**Figure 3.**
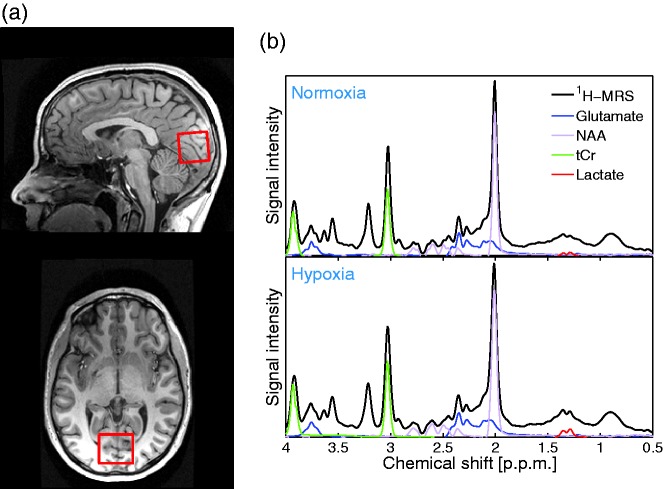
(a) Location of MR spectroscopy voxel (voxel size=30×35×30mm3) used to measure various metabolite concentrations. The voxel is located in the visual cortex in the occipital lobe covering the calcarine fissure. (b) Example of spectra measured during normoxia and hypoxia. The peak used for quantifying N-acetylaspartate (NAA), glutamate, lactate and total creatine concentrations (tCr) are visible.

Magnetic resonance spectroscopy was performed to measure the concentration of total creatine (tCr), i.e. PCr and Cr; NAA; lactate and glutamate. A water-suppressed point-resolved spectroscopy (PRESS) pulse sequence was used (TR=5000ms, TE=36.5ms, voxelsize=30×35×30mm3, 64 acquisitions, total duration 6min34s). The water signal was also measured and used as an internal standard for quantification.^22^ The voxel was located in the visual cortex covering the calcarine fissure (Figure 3(a)). The entire voxel was located inside brain tissue to avoid signal from subcutaneous fat, which has the same frequencies as lactate and could potentially confound the quantification of the lactate peak. Example of spectra measured during normoxia and hypoxia can be seen in Figure 3(b). Two spectra were recorded during normoxia and two spectra were recorded during hypoxia, all with visual stimulation. Visual stimulation was provided via video goggles (NordicNeuroLab, Bergen, Norway, 800×600 pixels, refresh rate 85 Hz) presenting an 8 Hz flickering checkerboard to evoke maximal neuronal activity and stimulate lactate production.

Post-processing and quantification of the spectra were performed with LCModel (LCModel (Version 6.3-1F), Toronto, Canada). Absolute concentrations of metabolites were estimated using the unsuppressed water signal as the reference signal. Mean concentrations were calculated from the two spectra measured during normoxia and the two spectra measured during hypoxia.

### Anatomical scan

High-resolution anatomical scans were obtained with a 3D T1-weighted turbo field echo sequence (150 slices, FOV=241×180×165mm3, voxel size=1.09×0.81×1.1mm3, TE=2.78ms, TR=6.9ms, flipangle=9∘).

### Statistics

The significance of the effect of hypoxia on the measured parameters was calculated using a paired Student’s t-test (Table 1).

To test the effect of the degree of hypoxia on the measured parameters a mixed linear regression model was used. The measured parameter was modeled as the response variable, CaO_2_ was modeled as a fixed variable and subject grouping was modeled as a random variable to account for between-subject variability. CaO_2_ was used as the fixed parameter, as this is the total amount of oxygen available in the arterial blood and therefore indicates the degree of hypoxia in the tissue.

Both hypoxemia and hypocapnia are suspected to have an effect of the measured parameters. In order to test for the significance of each effect a similar mixed model but with both CaO_2_ and PaCO_2_ as fixed parameters were also calculated (Table 2).

**Table 2.** Summary of statistics from mixed linear model with CaO_2_ and PaCO_2_ as fixed parameters.

	CaO_2_ weight	CaO_2_ *p* value	PaCO_2_ weight	PaCO_2_ *p* value
Cerebral metabolism				
CDO_2_ (µmol/ 100 g/min)	−7.2	0.37	55.3	<10-5 ^a^
CBF (mL/100 g/min)	−10.5	<10-6 ^a^	6.9	<10-3 ^a^
A-V O_2_ (%)	0.03	<10-4 ^a^	−0.041	<10-4 ^a^
CMRO_2_ (µmol/ 100 g/min)	−8.8	0.007^a^	1.6	0.70
NAA (mmol/L)	0.073	0.21	−0.019	0.82
Glutamate (mmol/L)	−0.016	0.63	−0.18	<10-3 ^a^
Cerebral lactate (mmol/L)	−0.15	<10-6 ^a^	−0.31	<10-6 ^a^
tCr (mmol/L)	0.080	0.13	0.23	0.004^a^

a A statistically significant change (*p* < 0.05).

CDO_2_: cerebral O_2_ delivery; CBF: cerebral blood flow; A-V O_2_: arteriovenous O_2_ saturation difference; CMRO_2_: cerebral metabolic rate of O_2_; NAA: N-acetylaspartate; tCr: creatine and phosphocreatine.

To test for a possible effect of lactate concentration on CBF, a mixed model was used with CBF as the response variable; lactate concentration, PaCO_2_ and CaO_2_, as fixed variables and subject grouping as a random variable. Oxygen and CO_2_ are known agents in controlling CBF and are therefore included in the model. Lactate concentration, PaCO_2_ and CaO_2_ were orthogonalized by whitening transformation before entering the model to avoid co-linearity. The model therefore tests for an independent effect of lactate on CBF.

Statistical calculations were performed using the Matlab statistics toolbox.

## Results

During hypoxia, CaO_2_ decreased significantly (*p*<10-6) with 20.5% from 8.5 ±0.6 mmol/L to 6.8 ± 13 mmol/L. SaO_2_ decreased significantly (*p*<10-6) from 98.0± 0.5% to 78.4± 11.2%. PaO_2_ decreased significantly (*p*<10-6) with 62.2% from 14.1 ±1.4 kPa to 5.3 ± 13 kPa. SvO_2_ decreased significantly (*p*<10-6) from 68.5±7.6% to 48.7±11.0%. PaCO_2_ decreased significantly (*p*<10-6), by 24.3% from 5.5±0.5 kPa to 4.1±0.8 kPa.

Heart rate increased significantly, by 20.9% (*p*<10-6). Mean arterial pressure (MAP) did not change (*p* = 0.17). Hemoglobin concentration increased significantly, by 1.2% (*p* = 0.035). Blood glucose concentration decreased significantly, by 8.2% (*p*<10-4). Blood lactate did not change significantly (*p* = 0.38).

CDO_2_ decreases significantly (*p* = 0.01) with 12.4%. CBF increased near-significantly (*p* = 0.058) with 15.5% from 58.2±6.5 mL/100 g/min during normoxia to 67.1±22.8 mL/100 g/min during hypoxia. The arteriovenous O_2_ saturation difference (A-V O_2_) did not change between normoxia and hypoxia (*p* = 0.89). CMRO_2_ increased significantly (*p* = 0.035) with 8.5% from 148.9±40.3 µmol/100 g/min during normoxia to 161.6 ±53.7 µmol/100 g/min during hypoxia. The variability in CMRO_2_ increased during hypoxia.

NAA concentration showed no change between normoxia and hypoxia (*p* = 0.36). Glutamate concentrations increased significantly (*p*<10-4) with 4.7%, cerebral lactate increased significantly (*p*<10-6) with 180.3% and tCr concentration decreased significantly (*p*<10-3) with 15.2%.

**Figure 4.**
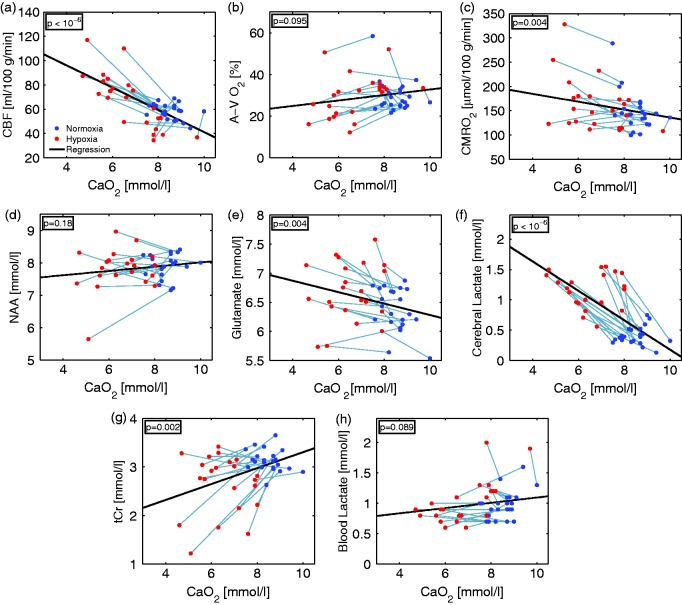
Correlations between arterial oxygen concentration (CaO_2_) during 10% FiO_2_ hypoxic challenge and (a) cerebral blood flow (CBF), (b) arteriovenous O_2_ saturation difference (A-V O_2_), (c) cerebral metabolic rate of oxygen (CMRO_2_); and concentrations of (d) N-acetylaspartate (NAA), (e) glutamate, (f) cerebral lactate, (g) creatine and phosphocreatine (tCr) in the brain measured with MR spectroscopy; and (h) lactate concentration in the blood.

Figure 4 shows the CBF, A-V O_2_, the calculated CMRO_2_, the metabolites derived from MRS and blood lactate with the corresponding arterial oxygen concentration. For one subject, the MRS measurement failed for technical reasons; this subject was excluded.

Figure 4(a) shows a significant negative correlation (p<10-6) between CBF and arterial oxygen concentration. However, for some subjects with high oxygen concentration during hypoxia, CBF decreased slightly.

Figure 4(b) shows no significant correlation between A-V O_2_ and arterial oxygen concentration (*p* = 0.095).

CMRO_2_ (Figure 4(c)) shows a significant negative correlation with arterial oxygen concentration (*p* = 0.004).

NAA concentration (Figure 4(d)) did not change with arterial oxygen concentration (*p* = 0.18). Glutamate concentration (Figure 4(e)) was significantly negatively correlated (*p* = 0.004) with arterial oxygen concentration.

Cerebral lactate concentration (Figure 4(f)) showed a significant negative correlation (*p*<10-6) with arterial oxygen concentration. In one subject, the lactate peak was not detectable during hypoxia, and this data point was removed from the analysis. The concurrently measured blood lactate (Figure 4(h)) level did not correlate with arterial oxygen concentration (*p* = 0.089).

tCr concentration (Figure 4(g)) was significantly positively correlated (*p* = 0.002) with arterial oxygen concentration. The results are summarized in Table 1.

Results from model separating the effects of oxygen and CO_2_ on the cerebral metabolism are summarized in Table 2. CDO_2_ was significantly positively correlated with PaCO_2_ (*p*<10-5) and not CaO_2_ (*p* = 0.37). CBF was significantly negatively correlated with CaO_2_ (*p*<10-6) and significantly positively correlated with PaCO_2_ (*p*<10-3). A-V O_2_ was significantly positively correlated with CaO_2_ (*p*<10-6) and significantly negatively correlated with PaCO_2_ (*p*<10-6). CMRO_2_ was significantly negatively correlated with CaO_2_ (*p* = 0.007) and not PaCO_2_ (*p* = 0.70). Glutamate was significantly negatively correlated with PaCO_2_ (*p*<10-3) and not CaO_2_ (*p* = 0.63). Cerebral lactate was significantly negatively correlated with CaO_2_ (*p*<10-6) and PaCO_2_ (*p*<10-6). tCr concentration (Figure 4(g)) was significantly positively correlated with PaCO_2_ (*p* = 0.004) and not CaO_2_ (*p* = 0.13).

Model testing correlation between CBF and decorrelated lactate concentration, PaCO_2_ and CaO_2_, showed significant positive correlation for PaCO_2_ (weight = 6.0, *p*<10-4) and lactate (weight = 2.8, *p* = 0.044) and a significant negative correlation for CaO_2_ (weight = –11.8, *p*<10-6). R^2^ for the model was 0.78.

## Discussion

The brain’s reaction to acute hypoxia is interesting from both a physiological and pathophysiological point of view, but comprehensive study of the brain’s reaction is difficult and often involves invasive measurements. However, by using combined MRI and MRS, fast and non-invasive measurements are now possible. To our knowledge, metabolite concentrations in the brain during hypoxia have not yet been measured using MRS.

We found evidence for metabolic changes, not only a CBF increase, during an acute hypoxic challenge in healthy young subjects.

### Blood gasses

At normoxia, the venous saturation was on average (68.5%) slightly higher compared to other studies using the same or different techniques, which normally reports saturations between 60% and 65%.^14,23^ We believe the higher values we measure could be caused by systematical bias from our scanner due to field inhomogeneity. We try to correct for inhomogeneities, however, a small bias could still be present. We believe the bias to be the same during normoxia and hypoxia, and therefore will not affect the result in a paired comparison.

**Figure 5.**
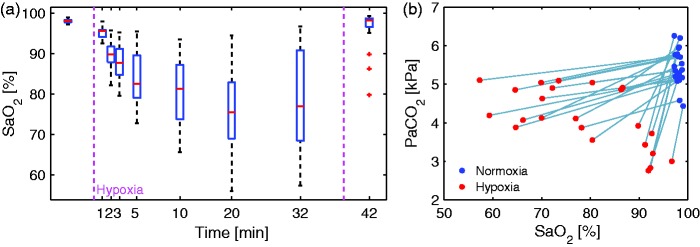
(a) Arterial saturation for each subject during 10% FiO_2_ hypoxic challenge. Large variability was observed between subjects. Some subjects seemed to be able to compensate almost completely for the inhalation of hypoxic air via pronounced hyperventilation and had almost no decrease in arterial saturation. By contrast, saturation in some subjects decreased to less than 60%. On average, the saturation decreased to 78% after 20 min of hypoxia, when the mean saturation reaches a plateau. (b) Arterial oxygen saturation and corresponding arterial CO_2_ pressure measured after 32 min of hypoxia. Subjects with a small decrease in arterial saturation had more pronounced hyperventilation and therefore a larger reduction in CO_2_.

The arterial oxygen saturation responses to hypoxia were heterogeneous (Figure 5(a)). Some subjects had an arterial saturation of more than 90% during hypoxia. By contrast, some subjects had a pronounced decrease in arterial oxygen saturation, to less than 60%. Subjects with high arterial saturation had the largest decrease of PaCO_2_ (Figure 5(b)), which we believe is due to different ventilator responses where these subjects had more pronounced hyperventilation.

We tried to differentiate between the effects of hypoxemia and those of hypocapnia by including both CaO_2_ and PaCO_2_ in a linear regression model. The Pearson’s r correlation between CaO_2_ and PaCO_2_ is 0.20, which we believe is low enough for CaO_2_ and PaCO_2_ to be included as independent variables in the model.

### Total cerebral blood flow

The observation of increased CBF during poikilocapnic hypoxia is in good agreement with published results. We found a negative correlation between CBF and CaO_2_ and positive correlation with PaCO_2_ as expected. Several studies have reported hypoxia-induced increases in CBF using different modalities.^1,2,5,6,24^ The standard deviation of the CBF was higher during hypoxia because of the subjects’ different ventilatory responses. By contrast, in subjects with high arterial saturation and low PaCO_2_ during hypoxia, CBF decreased slightly, probably due to hypocapnic-induced vasoconstriction (Figure 3(b)). Hypocapnic vasoconstriction during hypoxia has also been previously reported.^3,25^

### CMRO_2_

The effect of hypoxia on CMRO_2_ is less established than the effect on CBF and conflicting results have been published (summarized in Table 3). We found a significant increase of 8.5%. CMRO_2_ was significantly correlated with CaO_2_ and not with PaCO_2_. The global baseline CMRO_2_ measured was in good agreement with reported values obtained using different techniques.^5,26–28^ The standard deviation of our CMRO_2_ measurements increased during hypoxia, most likely because of the variable ventilatory responses.

**Table 3.** Previously published results of cerebral metabolic rate of oxygen, glucose and lactate during hypoxia.

Study	Findings	Number of subjects	Methods	Comment
Kety and Schmidt^1^	−CMRO_2_	7	Kety-Schmidt	10% FiO_2_
Cohen et al.^5^	−CMRO_2_	9	Kety-Schmidt	6.9–7.5% FiO_2_
	↑CMR_glu_			
	↑CMR_lac_			
Xu et al.^3^	↑CMRO_2_	16	MRI	14% FiO_2_
Smith et al.^4^	↑CMRO_2_	26	MRI	Stay at high altitude (3800 m)
Ainslie et al.^6^	−CMRO_2_	10	Doppler ultrasound; blood sampling.	90%–70% SaO_2_
	−CMR_glu_			
	−CMR_lac_			

− : denotes no change; ↑: denotes an increase.

The first study that examined CMRO_2_ during the inhalation of a hypoxic gas mixture was conducted by Kety and Schmidt,^1^ who found a non-significant 6% decrease in CMRO_2_ when subjects inhaled a 10% FiO_2_ gas mixture. A subsequent study using a similar technique found a non-significant 4% increase in CMRO_2_ during the breathing of 6.9–7.5% FiO_2_.^5^ A recent study on progressive hypoxia (90%, 80% and 70% arterial saturation) using invasive measurements also found no change in CMRO_2_.^6^

Recently, two MRI studies have reported a slight but significant 5.0% increase in CMRO_2_ during the inhalation of moderately hypoxic gas for 18 min (14% FiO_2_)^3^ and a significant increase in CMRO_2_ of 18% after a two-day stay at an altitude of 3800 meters (approximately 12.5% FiO_2_) and descent while breathing a hypoxic gas mixture.^29^ The method for measuring CMRO_2_ in these studies used an MRI technique that relied on the T2 relaxation of blood,^30^ in contrast to the method used in this study, susceptibility maps. These results indicate that our finding is independent of the MRI method used.

Differences in methodology, e.g. the variability of FiO_2_, make direct comparisons to other studies difficult. Severe hypoxia may yield a different response compared to moderate hypoxia. If the hypoxia becomes too severe, CMRO_2_ must decrease because of insufficient oxygen delivery to the blood and hence the brain.

### Magnetic resonance spectroscopy

#### Lactate metabolism

Studies have reported increased net lactate efflux from the human brain during hypoxia using tracer methods.^7^ Consistent with previous results, we found that acute hypoxia increased the concentration of lactate in the brain. We found correlation on increased cerebral lactate with decrease in both CaO_2_ and PaCO_2_. We did not observe changes in blood lactate concentration resulting from increased lactate production in the muscles, as observed in other studies.^7^ The reason for this lack of change is probably the limited muscle activity of the subjects, as they were lying motionless in the scanner when inhaling the hypoxic air. We corrected for the amount of blood lactate in the spectroscopic voxel and found a maximum contribution of 7%; therefore, peripherally produced lactate cannot explain the increase in cerebral lactate.

Lactate is an important energy source during hypoxic, ischemic or hypoglycemic injury and during stress and may have neuroprotective properties.^31^ In rat models, increased lactate levels are protective against excitotoxicity from increased glutamate levels.^32^ Increased lactate levels provide a source for fast energy production during increased glutamatergic synaptic activity. It has been shown that the neuroprotective effect of lactate is not mediated by improved glutamate uptake in animals.^33^

#### Glutamate

It is difficult to differentiate between the concentrations of glutamate and glutamine measured by MRS in 3T MR-scanner in humans in vivo. For this reason, the glutamate concentrations reported also includes glutamine concentrations. In addition, the spectroscopic level of glutamate does not necessarily relate to glutamatergic neurotransmission because extracellular and vesicular glutamate only represents a small fraction of the total amount of brain glutamate. It is likely that glutamate not encapsulated in vesicles is more MR-visible relative to its molar fraction. Therefore, the interpretation of the increased concentration is associated with some uncertainty, and studies should be repeated at higher field strength. However, glutamate is an excitatory neurotransmitter, and the increase observed during hypoxia could be an indication of increased neuronal activity. We found the increase in glutamate to primarily be correlated with decrease in PaCO_2_, which support previously published results on increased neuronal activity during hypocapnia.^34^

The increased glutamate concentration could result from reduced astrocyte activity and reuptake and a higher level of MR-visible glutamate in the synaptic cleft due to altered energy metabolism, as has previously been proposed.^35^

#### PCr and Cr

On average, we found a significant decrease in tCr; however, the subjects had heterogeneous responses. Some subjects showed a large decrease in concentration, whereas others showed no change. Increase in tCr concentration was primarily driven by decrease in PaCO_2_.

To our knowledge Cr concentration has not prior been measured in humans during hypoxia or hypocapnia. In animals studies the published effect of hypocapnia on Cr or PCr are diverse. Study has shown hypocapnia to decrease PCr in cats,^36^ on the contrary a study on rats found no change.^37^

Previous studies on hypoxia have found a decrease in PCr in animals.^38,39^ In moderate hypoxia, the decrease in PCr is not associated with decreased ATP concentration.^38^ However, in severe hypoxia with near-complete depletion of PCr, the ATP concentration also decreases.^39^

High Cr and PCr concentrations may have a neuroprotective effect against ischemic and hypoxic injury in animals.^40^ High PCr concentrations enable increased CK-mediated catalysis of ATP and could underlie the neuroprotective properties.^40^ Using 3T MRI in vivo in humans, it is not possible to differentiate between PCr and Cr concentrations measured by MRS. However, a shift towards increased CK-mediated ATP catalysis could potentially occur as a manifestation of hypoxia and a coping mechanism. Although these results are ambiguous, the change in tCr concentration is an indication of altered energy metabolism.

### Cerebral energy metabolism

In agreement with previously reported results, we found that during moderate-to-severe hypoxia in healthy humans, CBF increased and tended to compensate for hypoxemia.

In addition, we found significantly increased CMRO_2_, indicating elevated oxidative energy metabolism. It may seem counterintuitive to observe an increase in CMRO_2_ during hypoxia. However, even though oxygen delivery to the brain decreased slightly, the availability of oxygen in the brain is still sufficient to allow an increase in CMRO_2_. We speculate that the increased lactate concentration indicates further energy demand met by anaerobic glycolysis. An increased glutamate concentration could suggest increased neuronal stimulation or activity, which could be driven by the hypocapnia. Hypocapnia has been suggested as the reason for increased CMRO_2_ during poikilocapnic hypoxia by Smith et al.,^29^ who also reported increased CMRO_2_. The reported effects of hypocapnia on CMRO_2_ are diverse. A study of patients with ischemic cerebrovascular disease showed no conclusive result,^12^ and two studies on healthy humans reported a small but non-significant increase in CMRO_2_ during moderate normoxic hypocapnia.^41,42^ Hypocapnia has been shown to increase the excitability of neurons,^34^ which might be associated with the increased metabolic rate and glutamate concentration that we observed. Further investigation is needed to properly determine the effect of acute hypocapnia. The subjects in the study by Kety and Schmidt^1^ had mild hypocapnia compared to those in our study, and subjects in the studies by Cohen et al.^5^ and Ainslie et al.^6^ did not exhibit a decrease in CO_2_ pressure, which could explain the discrepancy in CMRO_2_ measurements.

We found the increase in CMRO_2_ to be correlated with decrease in CaO_2_ but not with PaCO_2_. However, all subjects had decreased PaCO_2_, and hypocapnia could still be a factor in the increase in CMRO_2_.

Increased energy metabolism could be associated with an increased cerebral metabolic rate of glucose (CMR_glu_) and lactate (CMR_lac_), as shown in a previous study of acute, 6.9–7.5% hypoxia in humans.^1^ Studies in rats have also found increased glucose transport^8^ and metabolism after sustained hypoxia.^43^ Hypocapnia has also been shown to increase CMRglu in rats^44^ and in humans during severe hypocapnia, but not in moderate hypocapnia.^42^ A recent study found no change in CMR_glu_ and CMR_lac_ in humans during progressive hypoxia (90%–70% SaO_2_).^6^

We found evidence for increased metabolic rate and neuronal activity. The reason for this is not obvious. For both hypoxia and hypocapnia there exist publications that indicate increased metabolic rate, with other studies indicating no change. The increased metabolic rate we observe could be a combination of the effects of increased excitability from hypocapnia and increased flow from hypoxia.

### CBF regulation

CBF is tightly coupled to arterial CO_2_ and O_2_ pressure. Lactate has also been proposed as a regulator of CBF, with effects mediated through astrocyte-induced vasodilation.^11^ We found, after decorrelation of the three parameters, significant correlation between CBF and PaCO_2_ and CaO_2_, but also lactate. This indicates that lactate concentration also has a regulating effect on CBF.

### Limitations

In these young, healthy individuals, ventilatory responses were highly variable. The large variation in ventilatory responses could result in different metabolic changes, making it difficult to find a decisive conclusion.

The measurement of global CMRO_2_ using MRI is a new technique, and few studies have been published, especially those that measure saturation outside of the normal range. A potential source of error is the partial-volume effect in voxels near the edge of the sagittal sinus. These voxels contain not only blood but also tissue, which will lower the susceptibility of the voxel. We corrected for this by finding the mode of the voxel values in the ROI, which is less sensitive to one-sided outliers than the mean.

Another potential error could result from magnetic field inhomogeneities. We corrected for this by fitting a thin-plate spline to the brain tissue surrounding the sagittal sinus and estimating the field inhomogeneities over the sagittal sinus. If there is strong variation across the sagittal sinus, this correction might not be sufficient. The inhomogeneities did not change substantially during the scanning sessions; thus, we believe that the potential error was constant during the normoxia and hypoxia measurements. We do not believe that the potential error was systematic between subjects.

Another potential source of error is the use of brain tissue as a reference value for fully oxygenated blood. During hypoxia, the susceptibility of the tissue would be expected to increase because of the less saturated arterial blood. To test whether this is a problem, we measured the susceptibility in an ROI in the brain tissue during the transition period from normoxia to hypoxia and found no systematic increase. If an increase in susceptibility in the tissue occurred, the calculated saturation in the sagittal sinus would be overestimated, and CMRO_2_ would be underestimated during hypoxia. Therefore, this possibility cannot be the reason for the increased CMRO_2_ measured during hypoxia.

The problems associated with the absolute quantification of brain metabolites using MRS have been previously discussed.^45^ As we compared the same subject between two situations, the problems with absolute concentration are mitigated. Hypoxemia in the brain will change the magnetic properties of the arterial blood and therefore will change the magnetic field in the scanner. This could potentially confound the measured spectra and could potentially explain the differences in the metabolite concentrations measured during normoxia and hypoxia. However, the NAA concentration did not change, which indicates the absence of a systematic error caused by changes in the magnetic field. The concentration of NAA is a marker of neuronal density and should not change from short periods of inhaling hypoxic air. In addition, the quantification of this metabolite was based on the area under the various peaks, which is relatively unaffected by a change in field strength.

## Conclusion

The present findings suggest that the response of the brain to acute poikilocapnic hypoxia includes not only an increase in CBF but also an alteration of energy metabolism.

We found increased cerebral blood flow and increased CMRO_2_, suggesting higher oxidative metabolism. We found an increase in lactate concentration and a decrease in tCr concentration, further suggesting an increase in metabolism. Furthermore, we found an increase in glutamate concentration, which may indicate higher neural activity.
